# Mesoporous Silica Nanoparticles as Drug Delivery Systems

**DOI:** 10.3390/ph18091392

**Published:** 2025-09-17

**Authors:** Flórián Benkő, Katalin Kristó, Tamás Sovány

**Affiliations:** Institute of Pharmaceutical Technology and Regulatory Affairs, University of Szeged, Eötvös u 6, H-6720 Szeged, Hungary

**Keywords:** mesoporous silica, nanoparticles, drug delivery, toxicity, functionalization, stimuli-responsive

## Abstract

Mesoporous silica nanocarriers (MSNs) have emerged as significant candidates in the pharmaceutical industry for drug delivery systems, suitable for a wide variety of drugs. Absorbing the active pharmaceutical ingredients (APIs) into the pores can be beneficial in several ways. The narrow pores may stabilize the APIs in an amorphous state, thereby improving its aqueous solubility and providing protection for the encapsulated drug against various factors in the human body, including enzymatic or chemical degradation, which enhances the bioavailability of the product. Beside the overview of their main characteristics, the present review focuses on the recent findings on MSNs form therapeutic and drug formulation perspective, including functionalization possibilities, as the size and surface of the particles influence the interactions with the cell membrane; therefore, the epithelial permeability of and release rate from the carrier, and may offer even targeted delivery in an organ-, tissue- or cell-specific manner, improving the therapy, also avoiding drug-related side effects.

## 1. Introduction

Nanocarriers can be divided into two main groups: organic and inorganic types. The organic group includes lipid nanocarriers, liposomes, polymeric nanoparticles, dendrimers, etc. In the case of the carriers in this group, the limited amount of delivered active pharmaceutical ingredient (API) is a clear disadvantage, in addition to reduced stability, and great sensitivity to temperature, pH, and enzymes in a physiological environment. The inorganic group includes metal oxide-, carbon-, silicon-, silica-, and quantum dot-based nanocarriers. Their advantages are greater stability and well-tunable physicochemical properties, but their accumulation in the body may be a crucial question of their applicability. Silica nanoparticles (SNP) are silicon dioxide (SiO_2_) nanoparticles in an amorphous state and have great potential in the pharmaceutical industry and drug development due to their special properties. According to pore size, the most common, most popular, and most effective type of silica nanocarriers are the mesoporous silica nanoparticles (MSNs) [1].

MSNs are SiO_2_ nanoparticles [2] with a pore diameter between 2 and 50 nm [3,4] and with a total diameter of a maximum of 1 µm. Their use in drug delivery dates to the early 1990s when they were synthesized for various scientific applications [5]. The material known as mesoporous silica was discovered by Mobil Oil Corporation in 1992 [6]. Their first drug delivery application was demonstrated in 2001 when MCM-41 silica was used to release ibuprofen [7]. Since then, MSNs have become an increasingly popular drug delivery system due to their advantageous properties and have been generally recognized as safe (GRAS) by the FDA. Small SNPs have been approved for human clinical imaging studies, setting the stage for their use in clinical applications of drug delivery. The number of related research activities is continuously increasing each year, expanding their role in cancer treatment, gene delivery, and oral disease therapy [5]. Their pore size is located between microporous and macroporous systems. They can be used in various shapes (sphere, cube, ellipsoid, rod) and sizes, with different pore sizes and pore structures [2,3,4,8,9,10]. They have a high degree of stability [8] and a rigid framework, which results in resistance to temperature, pH, enzymatic degradation, and mechanical force [4]. Thanks to the large number of pores, they have a huge specific surface area (approx. 700–1300 m^2^/g) and pore volume (approx. 0.5–1.5 cm^3^/g), which enables effective adsorption of various molecules [2,3,4,8,9,10]. By changing the pore size distribution, the range of adsorbable molecules can be controlled [3], which also enables separation based on size exclusion, which means selective adsorption of molecules of certain sizes. Depending on the size of the pores, small molecules as well as high molecular weight proteins can be transported into the pores [8]. In addition, chemical separation is also possible if the surface of the pores is coated with specific functional groups [4], which enables the separation of the APIs according to their structure [4]. Such modifications may include the setting of hydrophilicity/hydrophobicity of the surface, depending on whether the adsorption of hydrophobic or hydrophilic molecules is targeted [4]. By inserting different functional groups, the surface charge can also be controlled so that certain molecules will bind more efficiently. MSNs have a negative surface charge in biological environments, which promotes the electrostatic adsorption of positively charged APIs in the pores [10]. Furthermore, the biological markers attached to the surface of the nanoparticles enable the drug-carrying system to deliver the API in a tissue-specific manner [2,4,8,9,10]. A considerable problem is the premature release of the API from the pores during transport [4,8], which can be solved by using gatekeepers that respond to special stimuli and keep the pores closed until the stimulus [2,4,8]. In this way, controlled drug delivery and stimuli-responsive release can be achieved [2,4,9]. The drug release could be triggered by temperature, pH, redox potential, light, enzymes, magnetic field, or different ligands [4,8,11].

## 2. Effect of the Physicochemical Properties

The physicochemical characteristics of SNPs have a significant impact on pharmacokinetics, thus influencing distribution, accumulation in various cells, degradation, and elimination from the body [1,10]. It is also known that the particle size, particle morphology, pore structure, and surface properties are mostly to blame for possible toxicity, so ultimately also for the effectiveness of the therapy [10]. Furthermore, the adsorption of molecules into the pores can also be influenced by tailoring these properties [1,12]. The parameters of the particles can be changed within a wide range and can be customized according to the wishes [10,12]. The conditions of the synthesis can precisely tune and reproduce the particle size, particle shape, or pore structure, which can make drug formulation and therapy more effective [1,10]. Understanding the physicochemical properties of nanoparticles is crucial to achieve more effective treatment. The most important articles covered in this section are included in Table 1.

### 2.1. Particle Size

The size of the particles greatly affects the interaction with epithelial cells, cellular absorption, and thus their effectiveness [1,10,12]. In addition, the size also influences the leakage of the API [12]. It has been proven in vitro that epithelial permeability can be increased by reducing the size [1,12,13,24]. Zhang et al. also observed that the cellular uptake of MSNs occurred in a time-, concentration-, and size-dependent manner based on in vitro studies performed on Caco-2 cell lines (20 nm, 60 nm, 90 nm) [24]. Nevertheless, more complex results were obtained in vivo. Wang et al. compared differently sized nanoparticles in various aspects (100 nm, 250 nm, 480 nm) [12]. Similarly, Lamson et al. compared MSNs with different sizes to observe the effect of the particle size on bioavailability (20–1200 nm) [13]. Larger particles (generally around 500 nm) show slower release of the API and can form a strong interaction with the cell membrane [12], but cellular uptake is limited due to their large size [1,12]. Smaller particles (below 100 nm) show weaker interaction with the cell membrane but exert good cellular uptake due to their small size [12]. On the other hand, the smaller particles can slowly diffuse through the mucin layer covering the surface of the intestinal mucosa, become trapped in it, and aggregate, further worsening the absorption [1,12,13]. Furthermore, the decreasing particle size results in increased release rate, due to the larger specific surface area and faster degradation of the particles [1,12], which can lead to early leakage of the molecules, worsening their absorption [1,12]. As the diameter increases, the rate of degradation and drug release kinetics decreases, so larger particles have a longer half-life [12].

### 2.2. Particle Morphology

Silica nanoparticles can be produced and used in various shapes (sphere, cube, ellipsoid, rod). The shape of the particles can be characterized by the aspect ratio, which is 1 for spherical particles, but can reach 4 in the case of elongated rod-shaped ones [1]. Particles with different aspect ratios may exhibit different biodistribution and elimination [10,19,25]. The residence time in the gastrointestinal system is influenced by the particle shape [1,19]. Spherical nanoparticles have a shorter residence time than rod-shaped ones [19] and tend to accumulate in the liver. At the same time, in the case of rods, a more significant accumulation is observed in the spleen [10]. In addition, particles with a higher aspect ratio exert longer half-life in the bloodstream and show slower elimination [1,19], possibly due to faster renal excretion of spherical than rod-shaped particles [19,25]. By increasing the aspect ratio, a longer degradation time can also be observed [25].

### 2.3. Pore Size and Pore Structure

The carriers can have ordered and disordered pore structures [1,2]. The ordered mesopores have a regular, homogeneous pore structure and a tunable internal frame, which allows the optimization of the API formulation, while the disordered ones have a random distribution of pore structure. Pores can be divided into two main groups according to their geometry. There are open and closed pores, based on whether the pore is accessible to the medium or not. In the case of open pores, further differentiation is also possible. Blind pores are only accessible from one direction, while through pores are accessible from at least two directions [3]. The arrangement of the pores can also be defined and changed, thus the kinetics and dynamics of the release of the API can be influenced and regulated [1]. Laminar (tubular), radial, hexagonal, or cubic arrangements can also be created [1]. It is well known that the pore size significantly affects drug loading and drug release [1,3]. However, the literature is not crystal clear in terms of correlation [14]. Some studies show evidence that larger pores provide faster drug release [15,16,26], and larger pores have shown a faster degradation rate in the lymph nodes [15], and reports limited release for smaller pores as they limit the diffusion process, which is called the steric hindrance effect [16,26]. However, in other publications, evidence suggests that larger pores facilitate slower drug release, as the drug is loaded and trapped in deeper channels, requiring a longer access time for the release medium [14]. In general, the adsorption capacity can indeed be increased by increasing the specific surface area of the carrier and by decreasing the pore diameter [17].

### 2.4. Surface Chemistry

By connecting different functional groups, smaller or larger changes can be observed in the surface charge (zeta potential values) and hydrophilicity/hydrophobicity of SNPs [14,18]. Such functional groups can be, for example, -NH_2_, -PO_3_, -SH, -COOH, and -CH_3_ [14,18]. By functionalizing the internal surface (pores), the drug loading and the stability of the molecules can be increased, the release kinetics can be influenced, and even controlled release can be created [2,18,27]. By functionalizing the outer surface, the stability and biocompatibility of the particles can be improved [2,27]. The outer surface also affects the interactions with the cell membrane, cellular uptake, and penetration [2,12,18,27,28], and enables the creation of cell-specific targeted therapy [2,27]. The hydrophilic/neutral surface favors permeability through the mucus barrier, while the hydrophobic/cationic surface promotes penetration through epithelial cells [12,18]. Cationic nanoparticles have a higher degree of internalization than anionic particles [18,28]. Positively charged functional groups (-NH_2_) can establish electrostatic interactions with the negatively charged cell membrane, leading to increased endocytosis compared to negatively charged groups (-SH) [18,28]. SNPs also have permeation-enhancing properties as they can temporarily open epithelial tight junctions due to their size and surface, thereby increasing the drug absorption [13,14]. SNPs smaller than 200 nm with a negative surface charge have this property [13], while particles with a positive or neutral surface do not show similar effects [13].

## 3. Toxicity

Although MSNs can generally be recognized as safe products, their use during therapy may cause side effects in the body [1,2,29]. Due to their small size and large surface area, they may tend to accumulate in the body, or certain organs (liver, spleen, kidney, lung) and may even be toxic in higher concentrations, which is a clear disadvantage [1,4,27]. The silanol groups (Si-OH) on their surface can interact with the phospholipid layer of the cell membrane, thereby causing cell lysis or, in extreme cases, hemolysis [4,8]. In severe cases, the lack of hemocompatibility can also cause problems [2]. Overall, signs of cytotoxicity, haemotoxicity, and genotoxicity were also found [4,8]. That is why the biocompatibility of all similar preparations must be strictly controlled [2,4,27]. The physicochemical characteristics (particle size, particle morphology, surface chemistry, pore structure, etc.) of nanocarriers also have a major impact on toxicity [2,4,8,30]. In addition, further attention must be paid to the biological effects of their decomposition products, because they may also cause unwanted side effects [1]. The breakdown of silica nanoparticles in the body can take up to a few days. The initial hydration is followed by hydrolysis, and, finally, they are excreted in the urine in the form of silicic acid [1]. However, the literature is contradictory in this regard [31]. Some experiments show no considerable cytotoxic effect [18,31], while other experiments say otherwise [23,32]. For example, an in vitro study was performed with multiple concentrations, surfaces, and particle sizes (60 nm, 100 nm, 300 nm) of MSNs, with an exposure time of 72 h [18]. This study demonstrated no significant cytotoxic effect in any of the cell lines tested (human cervical (HeLA), epithelial lung (A549), and glioblastoma (U251)) [18]. Ismail et al. investigated the cytotoxic effect of silica nanoparticles in vivo in mouse models [33]. Amorphous silica nanoparticles were administered to the animals orally, at a dose of 150 mg/kg/day, for 3 months. Based on these mouse models, no toxic effects were detected under sub-chronic treatment of the nanoparticles [31]. In another study, particle size (15 nm, 60 nm, 200 nm) dependent impact was observed on cellular viability in all four cell lines tested (THP-1 derived macrophages, A549 epithelial cells, HaCaT keratinocytes, and NRK-52E kidney cells) [23]. Smaller particles caused notable toxicity on each cell line, after the exposure time of 24 h, in contrast to the larger particles, which showed no cytotoxic effect [23]. A similar result has been published to verify the toxic characteristic of mesoporous silica. In this in vitro study, the researchers investigated the effect of MSNs with an average size of 7 nm on cell viability at three different concentrations. After 72 h of exposure, only the highest concentration (62.5 µg/mL) caused a significant decrease in cell viability of human lung fibroblast cells. This toxicity occurred in a time- and dose-dependent manner [32]. In an in vivo mouse model, MacCuaig et al. presented coating-dependent effects after repeated intravenous administration of MSNs. Chitosan-coated MSNs induced minor organ changes and were the most biocompatible. Uncoated and especially PEG-coated MSNs (especially 2K PEG) moderately augmented pre-existing vascular or organ lesions, implying that PEGylation has the potential to augment chronic risks, while chitosan enables safer long-term use [34]. An in vitro study of Barguilla et al. exposed epithelial lung cells to bare, PEG-functionalized, or galacto-oligosaccharide-functionalized MSNs for eight weeks. There was no reported long-term DNA damage, but malignant-like alterations like anchorage-independent growth, increased migration/invasion, and secretome-facilitated tumor promotion were triggered by PEG- and GAL-functionalized MSNs. Naked MSNs did not cause such alterations and were determined to be safer [35]. Coating, in general, exerts a significant impact on the safety of MSNs. Uncoated MSNs are relatively innocuous; the most biocompatible coatings were achieved with chitosan, but PEG and carbohydrate functionalization can be hazardous in the long term, from vascular toxicity in vivo to tumor-promoting activity in vitro [34,35].

## 4. Drug Loading

Filling the pores with an API can be solved in several ways, which can be classified into two main groups: organic solvent-free and organic solvent-based methods [36,37]. The percentages of entrapment efficiency (EE%) and drug loading (DL%) are calculated based on the following equations (Equations (1) and (2)) [11,38,39,40,41].

(1) $$Entrapment\;efficiancy\;\left(\%\right)=\frac{Entrapped\;drug}{Total\;drug\;added}\times 100\;$$

(2) $$Drug\;loading\;\left(\%\right)=\frac{Weight\;of\;drug\;loaded\;in\;MSN}{Total\;weight\;of\;loaded\;MSN\;}\times 100$$

Loading the drug into mesoporous carriers can be beneficial since aqueous solubility may increase and a more favorable dissolution profile can be achieved during application, resulting in higher oral bioavailability [36,42]. The large specific surface area of the carriers favors amorphization, and the narrow spaces of the pores prevent crystallization [3,20,43], since if the pores are small enough, it is thermodynamically more favorable for the API to remain in an amorphous state, thereby stabilizing its state [20]. The amorphous state is associated with higher Gibbs free energy and a higher degree of molecular mobility than the crystalline state [3,36], resulting in increased solubility compared to the crystalline form [3].

Mesoporous silica nanoparticles (MSNs) are a versatile platform for the improvement of water solubility and bioavailability of poorly water-soluble drugs through the stabilization of the API in the amorphous state. Two outstanding mechanisms facilitate such stabilization: physical entrapment within nanoscale pores with a diameter less than the size of the critical nucleus. In this case, the ordered molecular packing necessary to develop crystals cannot advance. Furthermore, the unique interactions between the functional groups of APIs and the surface silanol groups of MSNs may immobilize drug molecules at or close to pore walls [44,45]. Bavnhøj et al. used celecoxib to demonstrate that when loading is performed under the pore-filling capacity (PFC) of the silica matrix, the drug will be physically stable and amorphous for at least 18 months of dry storage. PFC-loaded as well as near-PFC-loaded samples recrystallized, especially upon exposure to moisture, demonstrating the razor-thin margin between the level of loading and stability [46]. Although advantages are present, long-term stability is undermined by stress recrystallization. For example, α-mangostin began to recrystallize after 7–14 days at 25 °C and 95% relative humidity in MSNs with slightly broader pore size than the molecular size of the API, indicating that pore size must be finely tuned for efficient confinement [47]. Similarly, simvastatin had the longest amorphous lifespan when loaded into pores of 4.5 nm. Still, it exhibited rapid recrystallization in materials with bigger pore size, underlining the critical importance of pore size for the preservation of amorphous stability [45,47].

Physical and polymorphic stability also depend on the way of loading. Prednisolone underwent a polymorphic transformation from form I to form II within pores when it was loaded into MSNs with the solvent-evaporation method, whereas melt-loading retained the original polymorph. This means that contacts between the solvent, API, and silica, and the presence of residual solvent can template some polymorphic structures. Furthermore, confinement within thin channels stabilizes metastable polymorphs that would otherwise very quickly convert into the thermodynamically stable polymorph in the bulk. This offers an opportunity to selectively stabilize desired polymorphs by pore size [48]. Dwyer et al. showed that confinement in nanopores is capable of stabilizing metastable polymorphs or even forming new polymorphs not accessible in the bulk. For instance, fenofibrate crystallized in favored metastable states during confinement in mesoporous matrices, while other APIs formed nanoscale crystals of different thermodynamic properties. The above findings show that MSNs are not only stabilizers but also nanocrystallization chambers that can govern polymorphic selection and routes [49].

To combat degradation and recrystallization, strategies such as surface functionalization with hydrophobic silanes, pore filling with polymeric co-formers, and selection of solvent-free loading methods (e.g., co-milling or melting) have been demonstrated to counteract water adsorption, increase drug-matrix interactions, and avoid solvent-templated polymorph formation. Tuning of pore size, surface chemistry, and loading processes allows MSNs to enable better long-term amorphous API stability and regulated polymorphism, thereby facilitating enhanced performance of nanoparticulate drug delivery systems.

Several recent research works can support the previous finding and show that APIs in MSNs maintain their chemical structure, unaffected by covalent bonding during adsorption, storage, or release. Porras et al. observed consistent FTIR peaks for drug-MSN systems (albendazole loaded into SBA-15), confirming the preservation of functional groups [50]. Ditzinger et al. accomplished the stabilization of haloperidol and carbamazepine in their amorphous forms through adsorption into mesoporous silica, maintaining their chemical integrity [51]. PXRD and DSC analyses confirmed that the drugs remained amorphous post-incorporation without recrystallization for at least three months. Notably, the absence of new PXRD peaks and DSC melting endotherms during this period indicates there is no chemical degradation or alteration of the chemical structure [51]. Minecka et al. demonstrated that while aripiprazole transforms from crystalline to amorphous form upon adsorption into mesoporous silica, the chemical structure of the API remains unchanged [52]. To track the molecular state of the drug during and after amorphization, calorimetric, X-ray diffraction, and dielectric methods were carried out by the researchers to provide evidence. The findings suggest that the steric hindrance effect prevents recrystallization, thus preserving chemical structures as amorphous APIs revert to the original form [52]. Knapik-Kowalczuk et al. found that celecoxib in Syloid 244FP exhibits stable FTIR and NMR signals and intact molecular dynamics after amorphization, showing stabilization via physical interactions (steric hindrance and surface interactions without additional bond formation) [53]. Richter et al. converted itraconazole to an amorphous form in the presence of mesoporous silica, without altering its chemical structure, by the twin-screw extrusion method, as confirmed by consistent thermal and diffraction analyses [54]. Furthermore, Antonino et al. highlighted the transition of naproxen and ibuprofen from crystalline to amorphous when they were adsorbed into mesoporous silica (Syloid 72FP) [55]. This change is purely physical, with no chemical alterations to the drug molecules. Methods such as differential scanning calorimetry (DSC), X-ray diffraction (XRPD), and solid-state NMR verify that the APIs’ chemical structure of the APIs remains intact throughout and after amorphization. Drug adsorption onto the silica surface creates a stabilized amorphous state that resists recrystallization without changing the chemical bonds of the APIs. This stabilization stems from physical confinement and interactions such as hydrogen bonding or van der Waals forces with the silica, without any chemical reactions or degradation [55]. In summary, MSNs achieve API amorphization and stabilization through steric confinement and non-covalent adsorption, thereby preserving chemical structures, as verified by consistent data obtained by researchers.

### 4.1. Solvent-Free Methods

In the case of solvent-free processes, the main advantage is that it is not necessary to remove the solvent residue from the system at the end of the process, and the concentration of the API can also be controlled [36,37]. Techniques employed in this class include physical mixing, co-milling, and melt sorption methods. The simplest method is physical mixing [37], where fast drug filling is possible without special equipment. During direct mixing of the mesoporous carrier and the drug, the silanol groups on the surface of the silica bind the drug molecules. However, its application is limited because it can be used mostly for water-soluble molecules [37,56]. Another method is co-milling, during which the mixture of the API and the nanocarrier is milled together [36,37,56]. In the case of the melt sorption method, the mixture of the API and the carrier system is heated above the melting point of the API under continuous stirring, while the melt is absorbed into the pores [36,37]. After that, the system is cooled under reduced pressure, so that the molecules will be in an amorphous state in the pores [36,37]. However, this method cannot be used in the case of heat-sensitive materials, due to the considerable thermal decomposition [20]. Microwave radiation can also be used for melting [2,36].

### 4.2. Solvent-Based Methods

**Figure 1:**
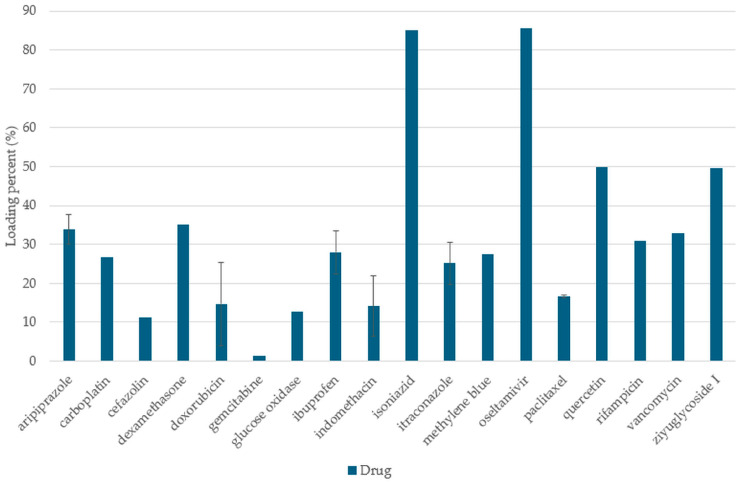
Achieved drug loading content for each API by the solvent immersion method, SD of the results are displayed if available.

Despite the advantages of solvent-free processes, solvent-based methods offer a more widespread alternative for filling the pores of the carriers. Each method listed here consists of two steps. First, mixing the drug solution and carrier, then removing the solvent [36]. The loading cycles can be repeated as many times as desired to increase the charging efficiency [20,36]. These techniques can be effectively applied to molecules both with good and poor aqueous solubility [37]. However, removing the solvent residue at the end of the process is essential, due to the toxicity of generally used organic solvents [56], and it is generally better to choose a less toxic organic solvent, if possible [37,56]. The adsorption of the API is also influenced by the properties of the solvent, such as viscosity or surface tension. This group includes the adsorption method, the incipient wetness approach, the solvent evaporation method, and the use of liquid and supercritical CO_2_. The adsorption method (solvent immersion method) is a low-energy process, but the pores can be filled effectively only if the API reaches a high concentration in the solvent [37]. The carrier system is immersed in the concentrated drug solution, while the liquid fills the pores due to capillary forces. The mesopores are then separated from the solution by filtration or centrifugation. Finally, the pores are dried under reduced pressure [2,36,37]. Drug loading capacities can be seen for some APIs by this loading method, in the following figure (Figure 1). During the incipient wetness impregnation, a highly concentrated drug solution is added dropwise onto the surface of the mesopores, in an almost equal amount to the pore volume of the carrier [37]. In this case, the pores are filled with liquid also due to the capillary forces. Then the pores are dried out at a higher temperature and under reduced pressure [2,36,37]. Nevertheless, the third method, solvent evaporation, is the most widely used because it can be applied to a wide range of molecules, regardless of their solubility, making it suitable for higher therapeutic doses as well. It is not necessary to prepare a concentrated solution since the concentration gradually increases during the process (due to the evaporation of the solvent). During the process, the drug should be dissolved in a volatile organic solvent, which is mixed with the carrier system [20]. All solvents are then removed by fast evaporation [37], while the drug is absorbed into the pores of the drug carrier [36,37]. The use of supercritical CO_2_ results in more efficient and deeper pore filling compared to other solvents. In this state, neither a liquid phase nor a gas phase exists. It combines the excellent diffusion capacity of gases and the excellent solvation capacity of liquids. In addition, it is non-toxic, non-flammable, and can be easily removed from the system. A cost-effective alternative can be liquid CO_2_, which is a similarly effective solvent [36,37].

## 5. Drug Release

It is necessary to understand the drug release mechanisms in porous media. Three categories can be distinguished based on the driving forces that create mass transport: advection, diffusion, and electrophoresis [3]. For nanocarriers, advection and diffusion are the most considerable types. In the case of advection, the liquid as a medium carries out the material transport. This fluid motion is ensured by mechanical forces (pressure, gravity, cohesive and adhesive forces) [3]. Furthermore, three subcategories can be distinguished here: film flow, capillary flow, and permeation. In the case of film flow, the liquid spreads into a thin liquid layer in contact with the surface of the pores. This spreading creates the required flow pattern. Capillary flow is ensured by capillary action. The surface tension of the liquid and the combined effect of adhesive and cohesive forces contribute to its creation. The pressure difference across the meniscus in the capillary causes the movement of the liquid along the capillary. During permeation, the flow of the liquid through the pores was created with the help of an external driving force (pressure) rather than an internal one [3]. Another significant material flow category is diffusion [2,57]. The driving force comes from the concentration difference, which is equalized by the Brownian motion of the particles [3].

According to some articles, the API is released from the pores according to first-order kinetics [2,57], but some experiments suggest that the release profile follows the Higuchi model [16,58], while other experiments suggest that the Korsmeyer–Peppas model describes the drug release rate most accurately [21,22].

Mathematical modeling, especially the application of the Fick diffusion equation, is very helpful in describing and predicting drug release from polymeric or porous matrices. Fick’s laws describe diffusion as the movement of molecules from a highly concentrated region to a region where the concentration is lower. Fick’s first law presumes the diffusion flux to be directly proportional to the concentration gradient. Fick’s second law controls the spatial and temporal change in concentration in the release medium [59]. For drug release, the general equation is as follows (Equation (3)):

(3) $$\frac{M_{t}}{M_{0}}\;=\;4\;{\left(\frac{Dt}{\pi h^{2}}\right)}^{\frac{1}{2}}\;$$

where *M_t_* is the amount of drug released at time *t*; *M*_0_ is the initial amount of drug; *D* is the diffusion coefficient in the matrix; and *h* is the thickness of the system.

This equation fits particularly well for premature release, up to 60% of the drug released, when there is no change in the matrix system during the release process. From a perspective of pore geometry, it essentially controls drug release rates by affecting effective diffusion pathways. Increased complexity of geometries increases tortuosity and reduces effective diffusion. High tortuosity and low pore connectivity reduce drug diffusion, consequently lowering the release rates. Additionally, surface roughness and pore size distribution affect surface area, diffusion, and residence times, and thereby drug release kinetics. Therefore, coupling the Fick diffusion model with parameters characterizing pore geometry (e.g., porosity, tortuosity, etc.) allows for more precise and automatic modeling of drug release kinetics. In this way, it is nicely explained why release profiles differ for chemically similar matrices due to differences in their geometrical shapes [59,60].

This supports that the release rate depends on several factors, among others, influenced by the pore size, the pore structure, the amount of drug loaded, the solubility of the API, and the resulting interaction between the carrier and the transported molecules [2]. By modifying these, the release kinetics can be controlled to a small extent, but a significant influence can only be achieved by surface functionalization of the mesopores. The following table contains some experimental data in terms of drug release in case of the previously shown APIs (Table 2).

## 6. Drug Administration

The present chapter discusses the most important findings in recent years regarding the utilization of different drug administration routes and methods.

### 6.1. Targeted Therapy

Targeted therapy, aiming, for example, at antitumor treatments, can be divided into active and passive forms [72,73]. Passive targeting is based on higher vascularity and higher vascular permeability of the tumor, as well as inflamed tissues, compared to physiological ones [73,74]. Furthermore, the lymphatic circulation of the tumor shows reduced efficiency [25], which resulted in easier accumulation of nanoparticles in the tumor or inflamed tissues [10,27,72,74]. This passive accumulation is generally called “ELVIS” (Extravasation through Leaky Vasculature and subsequent Inflammatory cell-mediated Sequestration) [72,74]. From the aspect of surface characteristics, negatively charged nanoparticles accumulate less effectively inside cells, due to the negatively charged cell membrane, which slows down the cellular uptake, so, in this case, a neutral surface charge is more favorable [25]. In contrast, active targeting can be achieved by functionalizing the outer surface of the silica carriers [10,27,72], where molecules are attached to the surface of the carriers that specifically bind to certain cell surface ligands and receptors, which are characteristic of tumor-, but not of healthy cells. This selective method provides more effective treatment and reduces the side effects of cytotoxic APIs on healthy cells and tissues [10,27]. The most recent findings on active targeting possibilities are summarized in Table 3. García-Fernández et al. prepared a mesoporous silica-based nanocarrier loaded with dexamethasone and capped with a specific protein to actively target the TNFR1 receptor, which is overexpressed in pro-inflammatory macrophages [72]. Since the carriers are preferably internalized by these macrophages, the cargo is released in a controlled manner, during enzymatic hydrolysis, thus preventing premature drug release, thanks to the gatekeepers. The targeting ability was tested in vitro with activated macrophages. On the other hand, the therapeutic efficiency was tested in vivo in mouse models. The researchers observed greater therapeutic effects and fewer adverse side effects than in the case of free dexamethasone. In conclusion, dexamethasone-loaded MSN has been successfully developed to actively target the lungs in acute lung injury (ALI), and this result may also be promising in other lung diseases (COPD, asthma) [72]. Teruel et al. presented a novel approach in oral inflammatory bowel disease (IBD) therapy, using functionalized magnetic mesoporous silica microparticles [75].

Magnetic particles were synthesized with an iron-containing coating. The model drugs were safranin O and hydrocortisone. The particles were functionalized with a bulky azo derivative to act as a gatekeeper and prevent early leakage of the drug. In vitro release models have demonstrated that most of the drug is released only in the colon, as desired. This is due to the reductive environment in the colon (the presence of the azoreductase enzyme), where the enzymatic degradation of the azo bond resulted in controlled release. In addition, in vivo pharmacokinetic studies were performed in a rat model of induced colitis to test the effect of the magnetic field during therapy. The results improved when the rats wore a magnetic belt, which extended the retention time of the particles in the desired areas [75]. Zhang et al. presented a study in which magnetic silica nanoparticles (filled with sulforaphane) were developed for the treatment of myocardial infarction [38]. In mouse models, effective accumulation was observed in the infarcted area after the application of an external magnetic field [33,75]. These results demonstrate the potential of using magnetic particles with magnetic field guidance [33]. Radhakrishnan et al. developed a drug delivery system (DDS) loaded with carboplatin for targeted lung cancer therapy [63]. The DDS was core–shell mesoporous silica functionalized with folic acid. Initially, the drug loading capacity of the functionalized carrier was more than twice as high per surface unit compared to the non-functionalized carrier due to the anchored -NH_2_ groups. The drug release was sustained but limited (up to 36%) in the case of the functionalized particle, similarly to previous cases. This is probably due to the stronger interactions between the amine groups and the carboplatin. The amino-functionalized particles were further functionalized with folic acid to increase cellular uptake of the particles, demonstrating the potential of folic acid-targeted therapy [63,65,76]. Cancer cells usually overexpress folate receptors, resulting in folate-mediated internalization that preferentially targets these cells [63,76]. This was demonstrated in a drug-mediated cytotoxicity analysis where amino- and folate-functionalized particles were evaluated and compared. Much greater cytotoxicity was observed in cells overexpressing folate receptors [63]. Similar research was carried out by Shirani et al., where folic acid functionalized MSNs were loaded with gemcitabine as a model drug, to investigate its potential in cancer treatment, while actively targeting cancer cells [70]. Results have shown that higher cellular uptake (HeLa and K562 cells) was observed in the case of the functionalized particles, compared with the non-functionalized carriers [70]. Jafarpour et al. demonstrated the importance of functionalizing the carriers to achieve targeted therapy using methotrexate as a model drug [76]. The particles were grafted with a copolymer to act as a pH-responsive gatekeeper and folic acid to actively target cancer cells [76]. Ortiz et al. similarly designed a pH-responsive DDS, loaded with the anticancer drug doxorubicin [11]. The particles were functionalized with transferrin through a pH-sensitive linker as a gatekeeper, controlling the release kinetics and preventing early leakage of the drug [11]. An interesting breast cancer therapy was published by Mal et al. using doxorubicin as API and mesoporous silica as a carrier [65]. The amine-functionalized MSNs were PEGylated and then conjugated with folic acid or hyaluronic acid. It was also published that coating the nanoparticles with polydopamine (PDA) and polyethylene glycol (PEG) leads to prolonged doxorubicin release from MSNs [77]. These conjugates enabled ligand-mediated targeted drug delivery [65]. Researchers have developed a successful DDS that targets cancer cells, thus avoiding the side effects associated with doxorubicin in conventional cancer therapy. This is important because in vitro cytotoxicity studies have shown that the free drug has higher cytotoxicity than the nanocarriers [65]. Shahbaz et al. developed a redox-responsive silica nanocarrier loaded with paclitaxel to target breast cancer cells [67]. The amine-functionalized silica core was coated with disulfide-functionalized non-porous silica shell. Then the core–shell carriers were further coated with polyethylene glycol via disulfide linkages. In vitro drug release assay verified the correlation between the redox potential of the environment and the release kinetics. This is an important finding, as it has been demonstrated that cancer cells possess a higher redox capacity than healthy cells. This provides controlled paclitaxel release for targeted therapy. According to cell uptake and cell viability assays, this DDS is noticeably more effective than free paclitaxel, making this nanocarrier an exciting prospect [67]. These examples highlight the importance of surface functionalization and using stimuli-responsive gatekeepers to achieve efficient targeted therapy.

Although the advent of active and passive targeting, particularly stimulus-responsive gates, represents an ideal marriage of the latest advancement and actual usage, e.g., folic acid and pH-responsive systems, a reference to likely challenges involved would be appropriate.

Nanoparticles (NPs) would naturally encounter some challenges in accessing ideal targets. They can be deposited in non-target tissues due to physiological barriers, heterogeneity of receptor expression, and defective targeting mechanisms, and therefore become potentially toxic and less effective [80]. In addition, environment-sensitive systems responsive to environmental stimuli such as pH may be activated before their intended use, or in normal tissues if the same environmental circumstances are present, leading to unwanted release of the drug [81]. Biological heterogeneity of patients, i.e., immune components such as anti-PEG antibodies, may influence the biodistribution of nanoparticles and off-target interactions as well [80].

The immune activation through functional nanocarriers is realized by interactions with innate immune cells such as macrophages, neutrophils, and complement proteins. Immune recognition triggers inflammation, immune responses, or even swift clearance, thus presumably constraining therapeutic effects. Surface properties, such as chemical composition, topography, and charge, may influence the immune cell activation and binding specificity. The possible immune interactions need to be well characterized for the development of stealth and immunocompatible nanoparticles [82]. Immune-derived side effects, such as cytokine release and complement activation, potentially corrupt the safety and the effectiveness of drug delivery [80]. Other issues that may affect nanoparticle penetration and the effectiveness of targeting are the heterogeneity of the target tissue and tumor microenvironment. Issues of stability, biodegradation rates, and nanoparticle agglomeration may also affect the effectiveness of action [80].

### 6.2. Advantages of MSNs in Various Administration Routes

The most recent findings and developments of MSN-based therapeutic approaches are displayed in Table 4.

#### 6.2.1. Dermal Drug Administration

The skin is our largest organ, which consists of three layers: the epidermis, dermis, and hypodermis. Due to its large surface area, it also serves as an important gateway for drug administration [25], which is suitable for both local (dermal) or systemic (transdermal) therapy [25,56]. Despite the popularity of this administration route, it is a challenge to get the API into deeper layers of the skin. The outermost layer of the epidermis, the stratum corneum, is the biggest barrier to molecular penetration [25,56]. However, the molecules must be absorbed through the capillaries of the dermis to achieve a systemic effect, which can be facilitated using nanocarriers [56]. In addition, sweat glands or hair follicles can be effective alternative routes for the molecules to enter the deeper layers of the skin [25,56]. In summary, it can be called as the transappendageal route. Among physicochemical properties, particle size is one of the most important factors influencing absorption. Particles in the range between 200 and 300 nm are suitable for local treatment of the skin in diseases such as atopic dermatitis [25] since particles larger than 75 nm do not pass through the stratum corneum [25]. Smaller particles (approximately 50 nm) are suitable for systemic treatment [25], and in general, it can be said that nanoparticles with a diameter smaller than 25 nm can penetrate the skin most effectively, so they can be used for transdermal drug administration with a suitable formulation [56]. By controlling the size of the nanocarrier, they penetrate to different depths in the skin. From the aspect of surface characteristics, cationic amino-functionalized MSNs show more efficient penetration into deeper layers of the skin than the particles with anionic surfaces of the same size [25]. The use of MSNs as a drug carrier may improve aqueous solubility, stability, and skin penetration of the drug [61]. In a research, gels containing MSN were prepared by using distilled water to form the base, adding the gelling agent (Poloxamer 407), and adding HPMC to enhance the viscosity [83]. In another publication, MSN containing oleogels were prepared with sunflower oil and the addition of colloidal silicon dioxide as a gelling agent [61]. According to in vitro experiments, the gel matrix also affected the drug release kinetics [61,83]. Slower drug release was observed in the case of gels containing loaded MSNs, compared to the free carriers, and the results showed close to zero-order release kinetics [61]. According to in vitro biocompatibility studies, this gel complex did not show any evidence of cytotoxicity on dermal fibroblast cells. Moreover, according to in vivo dermal safety studies, it has been proven that this preparation does not cause skin irritation during application, and so this formulation can be considered a safe product [61].

#### 6.2.2. Pulmonary Drug Administration

MSNs are also suitable for pulmonary drug delivery. Inhalation is an attractive route of administration since the application is non-invasive and patient-friendly [18]. However, the fast mucociliary clearance and the mucus barrier complicate the intake of the API and are obstacles to be overcome [18,25]. The mesoporous drug delivery systems may be very useful in the treatment of lung diseases, as they can passively accumulate in the alveoli [25,56,72]. The passive accumulation is due to their small size, while the large surface area, high vascularity, and vascular permeability of the lungs enhance the process [56]. Furthermore, targeted treatment of tumors or inflamed cells can also be implemented with the functionalization of their surface [56,72]. MSNs are usually delivered to the lungs in powder form with dry powder inhalers or suspended in liquid droplets with a nebulizer [56].

Ho et al. employed the Multiple-Path Particle Dosimetry (MPPD) model for aerosolization and determination of lung deposition of silver nanoparticles, which method may be extended to other materials like mesoporous silica nanoparticles [87]. It suggests particle size (mean 18 nm), density, and generation method as main factors that determine aerosolization efficiency and inhaled dose. Simulations of airflow, diffusion, sedimentation, and impact through airway generations yield comparable alveolar deposition fractions for particles less than 100 nm in humans and rats. With the addition of species-specific parameters, such as respiratory rate, lung surface area, tidal volume, and completed with possible lobar- and airway-level analysis, the MPPD model makes realistic regional retention predictions. Given that mesoporous silica nanoparticles are of similar size and physicochemical properties, the same methodology can estimate their aerosolization and lung deposition. In summary, the MPPD model is a useful tool in the assessment of inhalation hazard and in guiding the formulation of safer nanomaterials [87]. In another study, Ali et al. applied the MPPD model to simulate aerosolization and lung deposition of mesoporous silica nanoparticles [88]. Pre-characterized MSNs with pre-specified aerodynamic diameters were initially assessed through scanning mobility particle sizing to yield accurate size-distribution inputs. Asymmetric mouse airway geometry and physiological parameters such as respiratory rate, lung volume, and airflow were used in simulations that were approximated to provide a total lung deposition of about 45%, of which 37% was in the head, 27% in the tracheobronchial, and 36% in the pulmonary (alveolar) region. These predictions are in reasonable agreement with earlier in vivo results, validating the model. MPPD analysis also indicated that smaller MSNs exhibit more alveolar deposition, with the larger particles being better in the upper airways. Through linking aerosolization efficiency (via size distribution and concentration) and regional deposition, this modeling approach orients optimization of nanoparticle size and generation methods toward maximizing targeted lung delivery [88]. To achieve easier production of aerosols, the surface of nanoparticles is often provided with a polymer coating, thereby stabilizing their distribution and increasing dispersibility [56]. By reducing the particle size, deeper lung areas can be reached [25]. Particles smaller than 1 µm are capable of accumulating in the alveoli, but the ideal size range to achieve maximum efficiency is in the 200–500 nm range [25]. Nevertheless, another review mentioned that particle size in the range of 50–200 nm is ideal for maximizing drug localization by inhalation [89]. It has also been suggested that the ideal diameter is approximately 500 nm for phagocytosis by alveolar macrophages [89]. Li et al. nebulized PEI-PEG-coated MSNs (50 nm particle size) by standard nebulizers without compromising their structure, while keeping particles in their individual form within the water droplets [90]. The aerosol provides convenient delivery of MSNs to all areas of the respiratory tract—nasal, tracheobronchial, and pulmonary—and they persist for a week following inhalation, primarily within alveolar macrophages. It offers intracellular targeted delivery of lung diseases without passing through the gastrointestinal and first-pass metabolism. Despite decreased deep lung deposition in mice under nasal breathing, exposure is comparable to that of humans with inhalation. Toxicity tests showed no tissue damage or inflammation, further supporting MSN inhalation as a safe and effective method for extensive treatment of pulmonary diseases, feasible for clinical translation [90]. A study investigated the effect of surface chemistry on drug loading capacity, release kinetics, and antibiotic activity in pulmonary infections on *S. aureus* bacterial cultures [18]. The model drugs were isoniazid, rifampicin, and vancomycin. Remarkable absorption capacity was observed only with thiol (-SH) grafted carriers for all the tested drugs [18]. The in vitro drug release study showed sustained, but limited release: the cumulative release of rifampicin was 78.5% after 12 days, estimated by antibiogram [18]. Interestingly, the MIC values of the MSN-drug complex were higher for the free drug, probably due to the delayed and incomplete release from the pores [18]. Wang et al. developed a polydopamine-coated MSN formulation co-loaded with Ziyuglycoside I and Oseltamivir for the treatment of viral pneumonia. Interestingly, in vitro and in vivo studies have shown that this carrier provided immediate release of Oseltamivir (allows rapid virus killing) but prolonged release of Ziyuglycoside I (reduces the inflammatory response for a longer period) [62].

#### 6.2.3. Oral Drug Administration

Oral route is one of the most reliable and popular ways of drug administration, as it is one of the simplest and most convenient ways for self-medication [25,56]. The gastrointestinal system provides an excellent absorption surface for the introduction of the API into the systemic circulation [25]. However, in the case of molecules with poor water solubility and poor permeability, drug formulation involves many difficulties, which can be solved by using nanocarriers [42,56]. Ndayishimiye et al. proved that vancomycin, a high molecular weight and hydrophilic antibiotic macromolecule with poor oral bioavailability, can be effectively encapsulated using silica nanocarriers, and therefore, the necessary oral bioavailability can be ensured to eliminate the injection-related side effects [14]. The in vitro study showed a prolonged drug release from the nanocarriers compared to the pure drug form, and the in vitro permeability through Caco-2 cells showed a significant increase in the permeation for the encapsulated drug compared to the unencapsulated form [14]. These nanocarriers may also protect pH-sensitive molecules from the acidic environment of the stomach, thereby avoiding unwanted chemical changes, and in addition, they may also protect macromolecules from enzymatic degradation [25,56]. Based on various experiments, several key factors greatly influence the effectiveness of the therapy. The optimal size range was established between 50 and 200 nm [13]. Particles smaller than this are trapped in the mucus layer, and larger ones cannot pass through the tight junctions [13]. Anionic surface increases enteral permeability [13,14]. Overall, enteral bioavailability can be improved at several points.

Another study focused on repaglinide, an oral antidiabetic drug [84] from the BCS class II, with poor solubility but high permeability. In addition, it has a significant first-pass effect, and the combination of these two characteristics results in low oral bioavailability. The researchers prepared a repaglinide solid dispersion using mesoporous silica to improve solubility and drug release. Based on in vitro tests, the repaglinide-MSN complex exhibited significant improvement, compared to the pure drug [84]. Then this solid dispersion was successfully incorporated into a medicated chewing gum. This buccal administration causes the drug to bypass first-pass metabolism, further improving oral bioavailability. Based on their clinical investigations, the researchers proved that this formulation showed an increased antidiabetic activity compared to the marketed product by measuring blood glucose levels. In conclusion, this solid dispersion chewing gum formulation can be a promising perspective in antidiabetic therapy [84].

Mesoporous carriers also offer an attractive alternative in the oral therapy of macromolecules instead of the more complicated parenteral administration. Peptides and proteins may denature in the highly acidic environment of the stomach, lose their structure and function [1,13], while the proteases in the digestive system break down the protein chains, making the molecule useless and the therapy ineffective [1,13,91]. In addition, poor permeation through the epithelium is another hindering factor during the oral administration of macromolecules [1,13]. The macromolecule locked inside the pores remains protected against enzymatic or chemical degradation, and its permeation through the epithelium can also be improved [1,13,91]. It is also true that smaller pores provide better protection for macromolecules against degradation [1]. Mesoporous silica nanocarriers have additional interesting advantages due to their special properties that make them outstanding over other carriers and therefore offer a popular alternative in formulating macromolecules [1,13].

## 7. Oral Dosage Forms

### 7.1. Tablets

Tablets are the most used solid dosage form due to their numerous advantages. They are characterized by excellent physical and chemical stability, high patient compliance, and efficient economic production [92]. The incorporation of silica nanocarriers in tablet production presents a modern and innovative solution to various challenges, enhancing both the manufacturing process and therapeutic effectiveness. One of the significant benefits of mesoporous silica carriers is their ability to stabilize the API in an amorphous state, which greatly improves its solubility [30,39,42,43,92]. This enhancement leads to a considerable increase in the dissolution rate of tablets, thereby improving bioavailability and resulting in more effective therapy [30,39,42,92]. Additionally, nanocarriers can effectively mask the bitter taste and unpleasant odor of the API, potentially further improving patient compliance [42,93]. Zhang et al. observed a significant improvement in oral bioavailability with Telmisartan-loaded MSN carriers in vivo absorption studies conducted on dogs [24]. This MSN-containing tablet formulation was compared to commercially available tablets, with notable improvements attributed primarily to the enhanced permeability and solubility provided by the nanocarriers [24]. Furthermore, the physical and chemical stability of the API may also improve, which is particularly advantageous for sensitive molecules [39,92].

Certain molecules have an excessive adhesive property, and this represents another problem to be eliminated during direct tableting. In this case, a small amount of powder adheres to the surface of the upper- and lower punch or the die during each compression cycle [94]. As a result, tablet weight changes, and tablets with an uneven surface are obtained. More importantly, this may lead to poor content uniformity due to API loss. On the other hand, if the highly adhesive API is enclosed in the pores of the silica carrier, direct contact with the metal parts is eliminated. Thus, the loss of the API can be avoided [92,94].

However, mesoporous carriers are often associated with poor tabletability due to bad powder flow behavior; thus, direct tableting can only be achieved by adding various excipients in large quantities [95]. Such useful excipients include microcrystalline cellulose (filler), lactose monohydrate (filler), PVP (binder), sodium carboxymethylcellulose (disintegrant), croscarmellose sodium (super disintegrant), colloidal silica (anti-adhesion material), magnesium stearate (lubricant), and talc (lubricant) [39,95,96,97]. However, the drug content in the dosage unit decreases with high amounts of excipients added, so larger tablets must be designed to achieve the proper dose [95]. Another problem is that the greater compression force used during tableting may deform and damage the pore structure of the carrier, which might decrease the expected drug release rate [36,97,98]. When higher compression forces are applied, the pore diameter decreases, which affects both total pore volume and specific surface area, reducing the drug release rate from the carrier [97,98]. The use of microcrystalline cellulose may be a solution to this problem, as it deforms plastically under pressure while distributing the energy accumulated in the tablet during compression. Additionally, croscarmellose sodium may improve the drug release rate [97,98]. Granulation of mesoporous material is a proper choice to solve problems related to poor powder rheology.

The potential of MSNs may also be utilized well in the case of polypills. In this case, all drugs must be dissolved in the solvent. After that, the carriers must be loaded with this complex solution, using one of the already mentioned drug loading methods. This can be followed by tableting nanocarriers. This approach can be very useful in the treatment of hypertension, where combination therapy is most often used. Patient compliance could also be improved with the production of such a polypill, since taking one pill instead of multiple pills may be simpler for the combined treatment of high blood pressure [99].

#### 7.1.1. Low-Dose Tablets

One of the biggest challenges of tableting highly potent APIs (antibiotics, hormones, cytostatic molecules) is to achieve content uniformity [95,96]. Since these molecules are used in small doses, it is difficult to achieve a homogeneous powder mixture during mixing and to ensure this homogeneity until the end of tableting. Also, it is a challenge to develop a reproducible method that ensures content uniformity for each production batch, since the specified dose must be ensured between batches, not just within one batch [95,96]. A reliable alternative method is to use mesoporous silica as a carrier system instead of simple powder mixing, thus preventing the segregation and aggregation of the low-dose API during mixing and compression. In this case, the preloaded mesoporous carrier and the necessary excipients are mixed, and then the powder mixture can be compressed directly or after granulation. The drug loading can be controlled by the concentration of the drug solution used to fill the carrier systems, which affects the amount of the drug content in the final medicine [95,96]. Based on this, virtually unlimitedly low doses can be formulated into tablets, even in a concentration of 0.01%, just by properly preparing the solution [96]. The method is well-controllable and reliable, since each tablet has the same drug content and the desired content uniformity. Furthermore, the method can be used in both batch-based and continuous production [96]. This may be a promising finding because there is evidence that it is challenging to move from batch-based to continuous manufacturing while maintaining the desired drug content and content uniformity [94]. But it should be mentioned that in this study, no nanocarrier system was used [94], so using MSN formulations could make a huge improvement in developing continuous tablet manufacturing [96]. A study demonstrated that MSN carriers can also be used in orodispersible films to enhance the dissolution of poorly water-soluble drugs [100]. Based on in vitro drug release studies, more than 90% of prednisolone was released within 2 min when the loaded MSN was incorporated into the formulation. This means a much faster, immediate release in comparison with formulations containing the free form of the drug. In addition, precise dosing can be achieved using nanocarriers for personalized medication; moreover, enabling the production of low-dose preparations without content uniformity problems [100].

#### 7.1.2. Freeze-Dried Tablets

Lyophilization can be safely used for heat-sensitive drug substances because the drug is formulated at low temperatures and low pressure. Another advantage compared to direct compression is that the poor flowability does not cause problems here, and the pore structure of the nanoparticle is not damaged in the absence of compression. The freeze-drying process can be divided into three main steps: freezing, primary drying, and secondary drying [101]. The loaded carriers are homogenized with the prepared polymer solution [102,103], containing matrix agents (PEG, polyvinyl alcohol (PVA), hydroxyethyl cellulose (HEC), polyvinylpyrrolidone (PVP), alginate, dextran), cryoprotectants (sucrose, or sugar alcohols such as mannitol, sorbitol), surfactants (polysorbates, sorbitan monooleate), super disintegrants (croscarmellose sodium) [102]. The resulting dispersion is filled into blisters and then lyophilized [102,103]. During the process, the desired tablet shape is formed, adapting to the shape of the blister [102]. They are characterized by rapid disintegration (less than 30 s), low moisture content, and rapid dissolution [41,102,103]. Elmowafy et al. successfully optimized lyophilized tablet formulations using quercetin-loaded MSN carriers [41]. PVP-K30 was used as a polymeric stabilizer, PEG 6000 as a co-binding polymer, and sucrose as a cryoprotectant. This freeze-dried formulation allows fast disintegration and low friability (less than 1%). Importantly, the saturation solubility and the dissolution rate, and so the oral bioavailability of the poorly water-soluble quercetin improved notably, and it can be further improved by sublingual application [41]. Using the right composition, an orally dispersible tablet can be produced, which is why its sublingual use is advantageous, because the API goes directly into the systemic circulation (bypassing the enteral circulation, so the first pass effect does not apply), thus improving oral bioavailability [41,100]. In addition to immediate drug release, sustained and even targeted drug release is also available, provided by appropriate formulation, thus increasing the therapeutic possibilities. Nowadays, gastroretentive and mucoadhesive freeze-dried tablets are also available, further broadening the spectrum [102,103].

### 7.2. Granules/Pellets

Mesoporous silica nanoparticles have poor powder rheology and poor flow properties, due to their low bulk density, small particle size, and high hygroscopicity, which make further processing and formulation, especially with direct compression, difficult [104]. This problem can be eliminated by granulating the carriers. Flowability and compactibility can also be improved by granulation [86,104]. During granulation, the desired degree of dispersity is achieved by agglomeration of the particles, thereby creating an optimal flow characteristic. However, it is true that during granulation, the specific surface area and the pore volume of the carrier may decrease, also decreasing its adsorption capacity, which is a disadvantage from the aspect of the API absorption [104,105]. However, since this reduction is mostly negligible compared to the total specific surface area, a substantial improvement in its flow properties can considerably improve the overall performance [104,105]. The critical parameters for the process are the following: the amount of granulating liquid, the liquid addition rate, the mixing speed, the process time, and the temperature. Of course, the applied binder also plays an important role in the process [105]. These parameters affect the granule size, specific surface area, bulk density, flow properties (Hausner-factor, Carr-index), crushing strength, compressibility, and compactibility [105].

Wet granulation is the most frequently used technique to process MSNs, due to the many possibilities inherent in it [106]. Conventional wet granulation is well-suited for the granulation of silica nanocarriers, but several things should be considered during implementation. There are two possible sequences: first, the drug loading, followed by the granulation of the loaded carriers [104], or the opposite way, the granulation of the empty carriers, then loading the drug into the prepared granules. In the first case, during the planning and implementation of the process, special attention must be paid to the critical process parameters to avoid premature drug release [104]. In general, it can be said that as little moisture as possible should be used during granulation, thus reducing the risk of premature drug release. Still, at the same time, moisture sufficient to create the desired agglomeration must be ensured [104]. The risk of premature drug release depends to a certain extent on the chemical structure of the API and the carrier. Szewczyk and Prokopowicz developed doxycycline-loaded MSN pellets with prolonged release kinetics [40]. First, the drug was loaded into the pores, and then pellets were produced from the loaded mesoporous material with other excipients by wet extrusion and spheronization. For comparison, traditional doxycycline pellets were also prepared. This study demonstrated the importance of using mesoporous carriers for the delayed or sustained release of water-soluble drugs. Pelletization was a successful method to reduce the initial burst release of doxycycline; twice the release time was observed compared to traditional pellets [40]. Szewczyk et al. developed another DDS loaded with an antibiotic compound (cefazolin) for the treatment of osteomyelitis [66]. The amine-functionalized MSN was successfully loaded with the drug, and due to the strong interactions between the negatively charged cefazolin and the positively charged surface of the carrier, a prolonged release of 5 days was observed, which ensured a longer antibacterial effect than with classic pellets. This was proven by the microbiological activity assays of the pellets against *S. aureus* culture [66]. These results are promising in antibiotic or anticancer therapy, where therapeutic concentration is needed for a longer period [40,66]. A similar result was presented in another publication about metformin-containing MSN pellets with prolonged release kinetics [85]. After drug loading, pellets were formed with the mixture of the preloaded carriers and chitosan solution, and then the pellets were coated with five layers of chitosan. Since the stomach is not considered the optimal absorption surface for metformin, it is advisable to delay the release until conditions in the intestines are ideal for maximum bioavailability. Chitosan plays an important role in this formulation; it slows down and delays the metformin release and provides delayed and prolonged release kinetics, thereby preventing rapid blood level peaks after application [85]. On the contrary, Wang et al. published an article on MSN pellets filled with poorly water-soluble carbamazepine, and they observed better oral bioavailability compared to commercially available tablets based on in vivo analysis performed on dogs [86]. In vitro dissolution studies confirmed an increased dissolution rate compared to crystalline carbamazepine due to the amorphous state of the drug within the pores. This immediate-release pellet formulation allows for a reduction in the drug dose, maintaining the same clinical effect [86].

In the other case, the adsorption capacity of the carriers decreases due to granulation, which can impair the absorption of the molecules, since the binder can cover the surface of the pores or fill the channels [105,107]. In summary, the optimization of the granulation process is necessary for the drug-carrier complex used. Dry granulation cannot be used as a granulation technique for such carriers, as high compression forces can damage the pore structure [98].

To reduce or avoid the liquid used, it is possible to use other granulation techniques instead of the traditional wet granulation: either steam granulation or melt granulation, which reduces the risk of premature drug release compared to traditional wet granulation [98,106,108]. During steam granulation, steam is used instead of liquid water; it can be used with or without a binder. The steam shows a more uniform distribution than the atomization of the liquid; thus, more regular granules are formed [98,108]. The removal of water at the end of granulation can be achieved more quickly; a shorter process time is to be expected, so it is also an energy-efficient method.

In the case of melt granulation, it is necessary to use meltable excipients that can melt at low temperatures (50–90 °C) as a binder [98,108]. The molten binder forms the agglomerates during cooling and solidification. Since it does not require a liquid binder, it is advantageous for use in the case of moisture-sensitive molecules. However, its use should be avoided for heat-sensitive materials.

## 8. Conclusions

In recent years, mesoporous silica nanocarriers have attracted great interest in pharmaceutical science due to their advantageous properties. Simple synthesis, low production cost, and tunable physicochemical properties characterize them. Their behavior in the human body, including targeting possibilities, can be influenced by particle size, particle morphology, pore size, internal structure, and surface chemistry. By modifying the surface functional groups and the surface charge, targeted therapy is achievable in several medical conditions. A wide range of drug loading techniques is available (whether solvent-based or solvent-free methods), so the most appropriate method can be selected, and higher drug concentration can be encapsulated, improving loading efficiency. Nevertheless, their formulation for different delivery routes, such as dermal, pulmonary, or the most favorable oral administration, is extremely difficult due to their large specific surface, adhesivity, and low density, which results in poor powder flow properties, posing a huge challenge in the manufacturing and formulation of solid dosage forms. Agglomeration of the particles by granulation or pelletization is an excellent solution for improving flowability. Concluding this, the granulation process of mesoporous silica needs to be investigated and optimized to maintain the initially high porosity and high specific surface area of the material.

## Figures and Tables

**Table 1:** Experiments carried out to investigate the effect of physicochemical properties.

Particle Size (nm)	Functional Groups	Drug	Pore Size (nm)	Zeta Potential (mV)	References
100 250 480	Unfunctionalized MSN-PCD MSN-PCD-PEG	Fenofibrate	-	- +26.1 +5.44	[12]
20 50 100 200 500 1200	Unfunctionalized-COOH	Insulin	-	−57.6 −41.4 −41.2 −57.6 −68.9 −84.0	[13]
128, 152 232, 212 292, 158 779, 329	Unfunctionalized -NH_2_ -PO_3_ -CH_3_	Vancomycin	2 9	−26.33, −23.03 +32.67, +22.97 −34.1, −26.27 −17.83, −13.57	[14]
82.6 84.3 86.6	-	Antigen ovalbumin	7.8 10.3 12.9	approx. from −27 to −34	[15]
150 300	Unfunctionalized C8 C18	Ibuprofen, Erythromycin	3.6 5.7	-	[16]
-	-	Celecoxib Cinnarizine Paracetamol	2.5 7 17 21 24	-	[17]
60 100 300	Unfunctionalized -NH_2_ -SH	Rifampicin Vancomycin Isoniazid	-	-	[18]
278 289 250	Unfunctionalized -NH_2_ -COOH	-	2.5 2.3 2.4	−21 +30 −30	[19]
600 6000 11,000 35,000	-	Amlodipine Apixaban Deferasirox Ezetimibe Ibuprofen Lacosamide Valsartan	2.5 12.2 6.3 4.7	-	[20]
30,000–40,000	-	Ibuprofen	-	-	[21]
7500 3500	-	Artemether	2.9 16	-	[22]
15 60 200	-	-	-	−28.1 −30.6 −31.8	[23]

**Table 2:** The drug loading capacity and the drug release profile for each drug loaded by the solvent immersion method.

API	Drug Loading Content (%)	Duration	Released Drug (%)	References
Ibuprofen	30.01	45 min	100	[21]
	22.71			
	28.45			
	22.82			
Itraconazole	32.8	5 min	80	[58]
	21.9			
	25.1			
	21			
Doxorubicin	6.4	24 h	60	[11]
	6			
Quercetin	50	7 h	100	[61]
Ziyuglycoside I	49.6	72 h	82.51	[62]
Oseltamivir	85.5	24 h	82.86	
Rifampicin	31	24 h	68.5	[18]
Vancomycin	33			
Isoniazid	85			
Carboplatin	26.7	6 h	60	[63]
Indomethacin	10.57	1.5 h	100	[64]
	8.94			
Doxorubicin	15.13	168 h	87.5	[65]
	14.76			
	14.48			
	14.33			
Cefazolin	11.2	5 days	100	[66]
Dexamethasone	35	122 h	100	[38]
Paclitaxel	16.86	48 h	65	[67]
	16.87			
	16.13			
	16.72			
Ibuprofen	35.96	48 h	61	[42]
Doxorubicin	39.31	72 h	85.3	[68]
Methylene blue	27.37	72 h	51.5	
Doxorubicin	7.61	48 h	82.78	[69]
Glucose Oxidase	12.65			
Gemcitabine	1.49	168 h	77	[70]
Aripiprazole	29.7	1 h	69	[71]
	37.1			
	34.7			

**Table 3:** Experiments covering active targeting via surface functionalization.

Particle Size (nm)	Surface Functionalization	Specific Target Receptor	Drug	Treatment of	Pore Size (nm)	Zeta Potential (mV)	Refs.
100–200	pH-responsive linker (Transferrin)	-	Doxorubicin	Cancer	-	−22.8 (bare) −35.4	[11]
171	Magnetic surface (Fe_3_O_4_)	-	Sulforaphane	Myocardial infarction	2	-	[33]
88	palmitoyl (MSN-PALM) PEG- Phospholipids (MSN-PALM-LC)	-	Dexamethasone	-	2.4	−2.1 −6.3	[38]
100–250	-	Tumor necrosis factor receptor 1 (TNFR1)	Dexamethasone Rhodamine B	Acute lung injury (ALI)	2.5	−32 (bare) +7 +8	[72]
-	Magnetic particles (Fe_3_O_4_) coated with oleic acid	-	Hydrocortisone Safranin O	Inflammatory bowel disease	2.66	-	[75]
300–320	Folic acid	Folate receptors	Carboplatin	Lung cancer	2	−23.2 (bare) +36.3 (amine)	[63]
100–200	Folic acid	Folate receptors	Methotrexate	Cancer	3	−15.53	[76]
90–111	Folic acid (FA), hyaluronic acid (HA)	Folate and CD44 receptors	Doxorubicin	Breast cancer		−27.6 (bare) +2.09 (HA)	[65]
170–190	pH-responsive Carbon dots Folic acid	Folate receptors	Gemcitabine	Cancer	3.1	−16.2	[70]
125–200	pH- sensitive (polydopamine PEG)	-	Doxorubicin	Breast cancer	2.89	−19.43 (bare) −2.11	[77]
170–460	redox-responsive non-porous silica shell PEG coated	-	Paclitaxel	Breast cancer	2.42	−24.5 (bare) −10.4	[67]
210–245	pH/redox dual-responsive Bovine serum albumin Folic acid	Folate receptors	Doxorubicin	Breast cancer	-	approx. −28	[68]
190	Glycyrrhetinic acid	Glycyrrhetinc acid receptors	Chitosan oligosaccharide	acute drug-induced liver injury	6.15	−40.42 (bare) +7.013	[78]
66	pH-Responsive (polydopamine) Magnetic (Fe_3_O_4_)	-	Doxorubicin Glucose Oxidase	Pancreatic Cancer	5	−26.23 (bare) −14.24	[69]
180	Lipid coated	-	Docetaxel Tamoxifen	Breast cancer	2.27	-	[79]

**Table 4:** Experiments with different dosage forms and their potential use in treatment.

Particle Size (nm)	Pore Size (nm)	Zeta Potential (mV)	Route of Administration	Release Rate	Drug	Treatment of	Refs.
128, 152 232, 212 292, 158 779, 329	2 9	−26.33, −23.03 +32.67, +22.97 −34.1, −26.27 −17.83, −13.57	Oral	controlled Prolonged	Vancomycin	systemic MRSA infections	[14]
60 100 300	-	-	Pulmonary	-	Rifampicin Vancomycin Isoniazid	-	[18]
20 60 90	-	-	Oral Tablet	-	Telmisartan	Hypertension	[24]
1000–4000	7.34	-	Oral pellets	Prolonged	Doxycycline	bacterial infection	[40]
150	2.4	−12.3 to −29.0	Oral	Sustained	Ibuprofen	Musculoskeletal pain	[42]
100–250	2.5	−32 (bare) +7 +8	Pulmonary	Targeted Controlled	Dexamethasone Rhodamine B	Acute lung injury (ALI)	[72]
430–470	3.3–4.1	−27 (bare) −18	Dermal Transdermal Oleogel	Sustained	Quercetin	benign, malignant skin formations	[61]
-	-	-	Dermal Gel	Sustained	Rosmarinus officinalis extract	acute wound healing	[83]
760	10.9	-	Pulmonary	Immediate (OST) Sustained (ZgI)	Ziyuglycoside I Oseltamivir	viral pneumonia	[62]
-	4	-	Oral Chewing gum Solid dispersion	-	Repaglinide	diabetes mellitus	[84]
200,000–500,000	-	-	Oral pellets	Prolonged	Cefazolin	Osteomyelitis	[66]
220	6.1	approx. from −6 to −55 pH-dependent manner	Oral pellets	Prolonged	Metformin	type II diabetes	[85]
500–1500	6.5	-	Oral pellets	Immediate	Carbamazepine	Epilepsy	[86]

## Data Availability

No new data were created or analyzed in this study. Data sharing is not applicable to this article.
